# Microstructure and Tensile Properties of Cu-Ti Composites Deposited by Cold Spray Additive Manufacturing

**DOI:** 10.3390/ma18122787

**Published:** 2025-06-13

**Authors:** Jia Cheng, Jibo Huang, Haifan Li, Kejie Zhang, Haiming Lan, Hongmin Xin, Renzhong Huang

**Affiliations:** 1 China Yangtze Power Co., Ltd., Yichang 443002, China; cheng_jia1@ctg.com.cn (J.C.); li_haifan@ctg.com.cn (H.L.); 2 Guangzhou Institute of Hubei Chaozhuo Aviation Technology Co., Ltd., Guangzhou 510530, China; zhangkejie@cz-tec.com (K.Z.); lanhaiming@cz-tec.com (H.L.); xhm0330@163.com (H.X.)

**Keywords:** cold spray additive manufacturing, Cu-Ti composites, gas temperature, post-heat treatment, mechanical properties

## Abstract

In this study, copper–titanium (Cu-Ti) composite coatings with 6 wt.% titanium content were fabricated via cold spray additive manufacturing (CSAM) using nitrogen as the propellant gas. The synergistic effects of propellant gas temperatures (600 °C, 700 °C, 800 °C) and post-heat treatment temperatures (350 °C, 380 °C, 400 °C) on the microstructure and tensile properties were systematically investigated. Tensile testing, microhardness characterization, and fractography analysis revealed that increasing the propellant gas temperature significantly enhanced the plastic deformation of copper particles, leading to simultaneous improvements in deposit density and interfacial bonding strength. The as-sprayed specimen prepared at 800 °C propellant gas temperature exhibited a tensile strength of 338 MPa, representing a 69% increase over the 600 °C specimen. Post-heat treatment effectively eliminated the work-hardening effects induced by cold spraying, with the 400 °C treated material achieving an elongation of 15% while maintaining tensile strength above 270 MPa. Microstructural analysis demonstrated that high propellant gas temperatures (800 °C) promoted the formation of dense lamellar stacking structures in copper particles, which, combined with a recrystallized fine-grained microstructure induced by 400 °C heat treatment, enabled synergistic optimization of strength and ductility. This work provides critical experimental insights for process optimization in CSAM-fabricated Cu-Ti composites.

## 1. Introduction

Copper and its alloys are widely used in fields such as electronic devices, aerospace, and power and electrical engineering due to their excellent electrical conductivity, thermal conductivity, and good processing properties [1,2,3]. However, pure copper has relatively low strength, poor wear resistance, and poor corrosion resistance, which limits its application in some high-requirement environments [4,5]. To enhance the comprehensive performance of copper-based materials, researchers have developed composites reinforced with phases such as Ni, Cr, Ti, Al, W, etc. [6,7,8,9]. Among them, copper–titanium composites have received extensive attention because of their excellent wear and corrosion resistance [10,11,12].

As a solid-state deposition technology, cold spray enables high-speed metal particle deposition at low temperatures, effectively avoiding oxidation and phase transformation [13,14]. These advantages make it particularly suitable for copper-based composites, as demonstrated by recent studies optimizing bonding strength through particle velocity control [15,16].

Beyond copper, cold spray has expanded to hard-material coatings like Zr and Ti alloys. For instance, Ralls et al. utilized a high-pressure system (gas temperatures up to 1100 °C) to deposit dense Zr702 coatings on aluminum substrates, achieving oxide-free layers with enhanced wear resistance [17]. Similarly, Daroonparvar et al. reported that Ti coatings on magnesium alloys via high-pressure cold spray (HPCS) exhibited superior corrosion resistance due to controlled thermal softening [18].

The technology’s versatility is further evident in applications such as additive manufacturing and surface repair [14,19]. However, critical challenges remain: gas temperature, powder characteristics, and substrate state significantly influence microstructural and mechanical properties [16,19]. For copper-based composites, optimizing these parameters to enhance performance remains a key research focus [20].

At present, researchers have conducted extensive research on the preparation and performance optimization of cold-sprayed copper-based composites. Research shows that parameters such as gas temperature, powder particle size, and substrate roughness during the cold-spraying process have a significant impact on the density, bonding strength, and mechanical properties of the deposited materials [21,22,23]. Schmidt and Venkatesh et al. studied the deposition characteristics and bonding mechanism of cold-sprayed copper-based composites and pointed out that an increase in gas temperature can significantly enhance the degree of particle deformation, thereby improving the density and bonding strength of the deposited materials [23,24]. Huang et al. studied the post-treatment technologies for cold-sprayed copper-based materials and indicated that post-heat treatment can effectively eliminate the work hardening during the cold-spraying process and improve the ductility and mechanical properties of the material [25].

However, research on the cold-spray preparation of Cu-Ti composites and the optimization of their mechanical properties is relatively scarce. Existing studies mainly focus on composites such as Cu-Ni [6], Cu-Cr [7], and Cu-W [9], and there is a lack of research on the optimization of cold-spray process parameters and the influence of post-heat treatment for Cu-Ti composites.

In this study, a Cu-Ti (6 wt.% Ti) composite deposit was fabricated via the cold spray additive manufacturing technique, with nitrogen serving as the propellant gas. The effects of gas temperatures (600 °C, 700 °C, 800 °C) and post-heat treatment temperatures (350 °C, 380 °C, 400 °C) on the mechanical properties of the material were systematically investigated. Through tensile property testing and microstructure analysis, the regulatory mechanisms of gas temperature and post-heat treatment on the mechanical properties of the Cu-Ti composite were revealed. This research provides crucial experimental evidence for the process optimization of cold spray additive manufacturing of Cu-Ti composites.

## 2. Materials and Methods

### 2.1. Preparation of Cu-Ti Composite Samples

**Figure 1:**
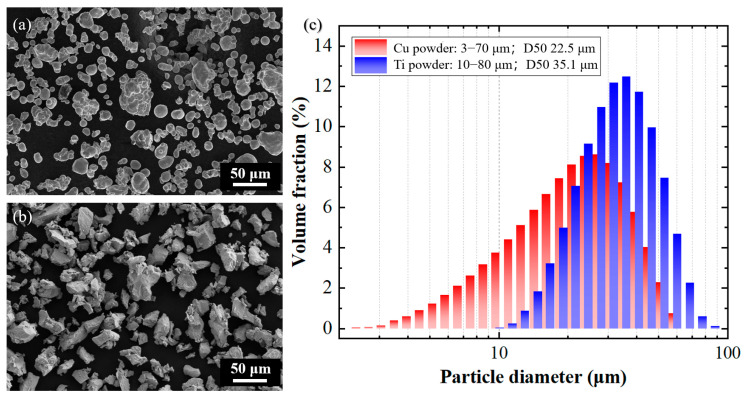
Powder characteristics for cold spray deposition of Cu-Ti composite materials: (**a**) microscopic morphology of Cu powder; (**b**) microscopic morphology of Ti powder; and (**c**) details of particle size distribution.

Commercial pure copper powder and pure titanium powder were used as raw materials. As shown in Figure 1, the copper powder particles had a nearly spherical morphology. The measured particle size of the copper powder ranged from 3 to 70 μm, with a D50 value of 22.5 μm. The titanium powder particles were irregularly shaped. The measured particle size of most of the titanium powder ranged from 10 to 80 μm, with a D50 value of 35.1 μm. The two powders were uniformly mixed by mechanical mixing according to the ratio of 94 wt.% copper and 6 wt.% titanium for the preparation of Cu-Ti composite deposits by cold spraying.

A commercial high-pressure cold spray additive manufacturing system (PCS-800, Plasma Giken Co., Ltd., Yorii, Japan) was employed to fabricate thick Cu-Ti composite deposits on the surface of sand-blasted stainless steel substrates. During the cold spray additive manufacturing process, pressurized nitrogen at 5 MPa was used as the propellant gas, with gas temperatures set at 600 °C, 700 °C, and 800 °C, respectively. These temperature parameters were selected based on systematic optimization: preliminary experiments indicated that gas temperatures below 600 °C resulted in insufficient particle deformation and poor bonding, while 800 °C represented the upper limit for stable long-term operation of the PCS-800 system. The detailed process parameters of the cold spray additive manufacturing process are as follows: the spraying distance was 30 mm, the spraying angle was 90°, the nozzle scanning speed was 400 mm/s, and the scanning interval was 2 mm. Coatings with a thickness exceeding 5 mm were deposited to meet the dimensional requirements of the tensile specimens. Block-shaped Cu-Ti deposits with dimensions of 150 mm in length, 100 mm in width, and 50 mm in height were prepared for subsequent microstructure characterization and mechanical property testing. After cold-spray deposition, the Cu deposits were separated from the substrate by electrical discharge machining (EDM) for material characterization and property testing.

To investigate the effects of post-heat treatment on the mechanical properties of Cu-Ti composite materials, the cold-sprayed deposited specimens were subjected to heat treatments at 350 °C, 380 °C, and 400 °C respectively. The heat preservation duration was maintained for 2 h, followed by furnace cooling to room temperature. The heat treatments were conducted in a box-type resistance furnace, with nitrogen gas introduced during the process to prevent material oxidation.

### 2.2. Mechanical Property Testing and Microstructure Characterization

**Figure 2:**
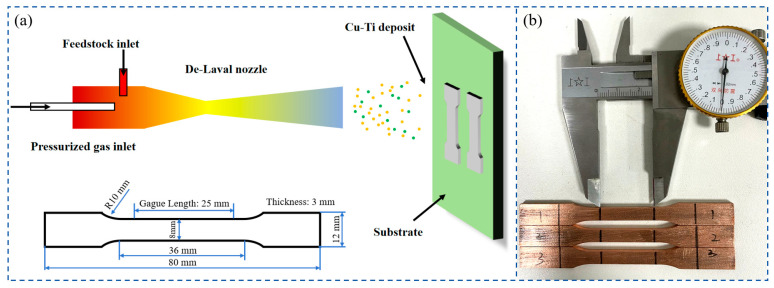
(**a**) Sampling configuration and dimensional specifications of tensile specimens extracted from cold-sprayed Cu-Ti composite deposits and (**b**) prepared tensile specimens.

The as-deposited cold-sprayed Cu-Ti composites and their post-heat-treated counterparts were precision-machined into tensile specimens using electrical discharge machining (EDM). Figure 2 schematically illustrates the specimen extraction protocol, with the tensile axis intentionally oriented perpendicular to the spray deposition direction to evaluate an isotropic mechanical response. The preparation of the tensile specimens refers to the previous research on the mechanical properties of the cold-sprayed copper deposits [25,26].

Quasi-static tensile tests were conducted under displacement control at a constant strain rate of 1 mm/min using a servo-hydraulic universal testing system (INSTRON 5982, Instron, Norwood, MA, USA). Three specimens were tested per material condition to ensure statistical significance. Key mechanical parameters, including ultimate tensile strength (UTS) and percentage elongation at fracture, were systematically recorded.

The Vickers hardness of the cross-sections of polished as-sprayed and heat-treated Cu-Ti composite deposit samples was determined using a Vickers Indenter (BUEHLER MICROMET5104, Akashi Corporation, Osaka, Japan). In each test area, five measurements were carried out to collect hardness data. The hardness tests were executed under a load of 1000 g with a holding time of 15 s.

An optical microscope (LEICA DMI5000M, Leica, Wetzlar, Germany) was employed to investigate the microstructure features of the as-sprayed and heat-treated Cu-Ti composite deposits. The polished samples were etched at room temperature for 15 s in an aqueous solution containing 5 g of ferric chloride (FeCl_3_), 10 mL of hydrochloric acid (HCl), and 100 mL of distilled water, after which the etched sample cross-sections were examined. Following the tensile test, a scanning electron microscope (GeminiSEM300, Carl Zeiss SMT Ltd., Cambridge, UK) was used to analyze the fracture surface morphology characteristics of the specimens. The phase composition of the Cu-Ti composite before and after heat treatment was analyzed by X-ray diffraction (XRD; Rigaku SmartLab SE, Rigaku Corporation, Tokyo, Japan) using Cu-Kα radiation operated at 40 kV and 40 mA, with a scanning speed of 2° min^−1^.

## 3. Results and Discussion

### 3.1. Microstructure

**Figure 3:**
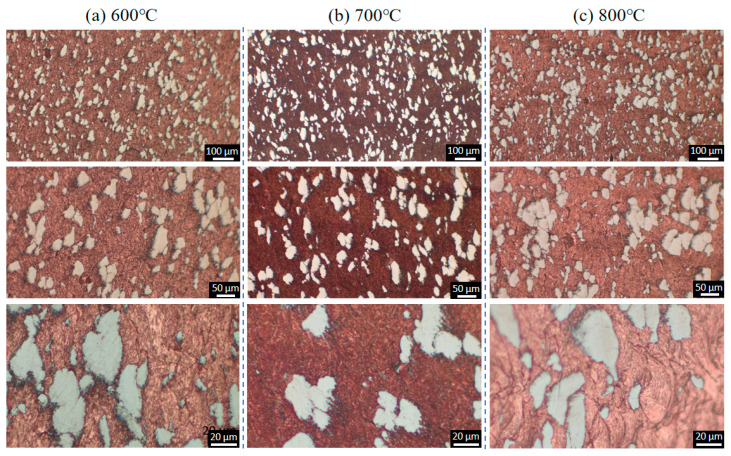
Microstructural evolution of as-sprayed Cu-Ti composite deposits under varying propellant gas temperatures: (**a**) 600 °C; (**b**) 700 °C; and (**c**) 800 °C.

Figure 3 presents the microstructures of the as-sprayed Cu-Ti composites fabricated through cold spray additive manufacturing at varying magnifications. The deposits prepared under the cold spray propellant gas conditions of 600 °C, 700 °C, and 800 °C all exhibit a dense structure, completely free of defects such as pores and cracks. Ti particles are uniformly and dispersedly distributed within the deposits, and there is no obvious deformation observed in these Ti particles. This phenomenon is likely attributed to the significant difference in hardness between Ti and Cu. Specifically, the hardness of Cu ranges from 35 to 45 HB, while that of Ti is 120 HB. During the spraying process, the Ti particles can be effectively encapsulated by the deformed Cu particles. Moreover, the impact energy of the Ti particles can be efficiently absorbed by the Cu matrix, preventing substantial deformation of the Ti particles. The volume fraction of Ti particles in the deposits under the three propellant gas temperature conditions is approximately 20%, showing no obvious differences in metallographic observations. This corresponds to the 6 wt.% Ti content in the as-mixed Cu-Ti feedstock powders.

From the enlarged views, it can be seen that the Cu particles in the deposits form a flattened, lamellar stacking structure due to intense plastic deformation, while the Ti particles are embedded in it in an irregular geometric shape. The connection between Cu and Ti phases is tight, with no interfacial defects present. The as-sprayed microstructure indicates that high-quality Cu-Ti composite deposits can be obtained under the cold spray parameters employed in this study.

**Figure 4:**
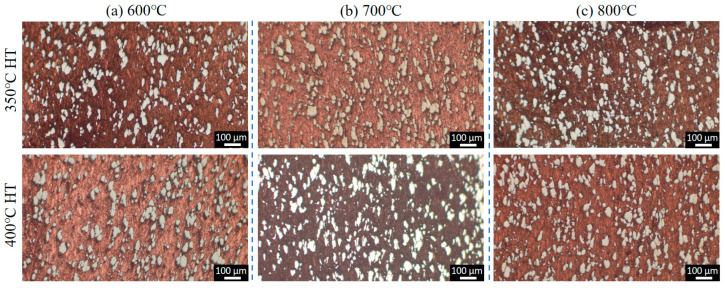
Microstructures of the Cu-Ti composite deposits prepared under different propellant gas temperatures after heat treatment: (**a**) 600 °C; (**b**) 700 °C; and (**c**) 800 °C.

Figure 4 depicts the morphologies of the Cu-Ti deposits after heat treatment at 350 °C and 400 °C. It is evident that following the heat treatment, the microstructure of the deposits has not undergone significant alterations. The Ti particles still remain dispersedly distributed within the Cu deposits in an irregular shape, and the interface between the Ti and Cu phases remains distinctly clear. Quantitative metallographic analysis reveals that the content of Ti particles is comparable to that in the as-prepared state. These results indicate that under the post-heat treatment temperatures employed in this study (ranging from 350 °C to 400 °C), there is no pronounced diffusion of elements between the Ti and Cu particles.

**Figure 5:**
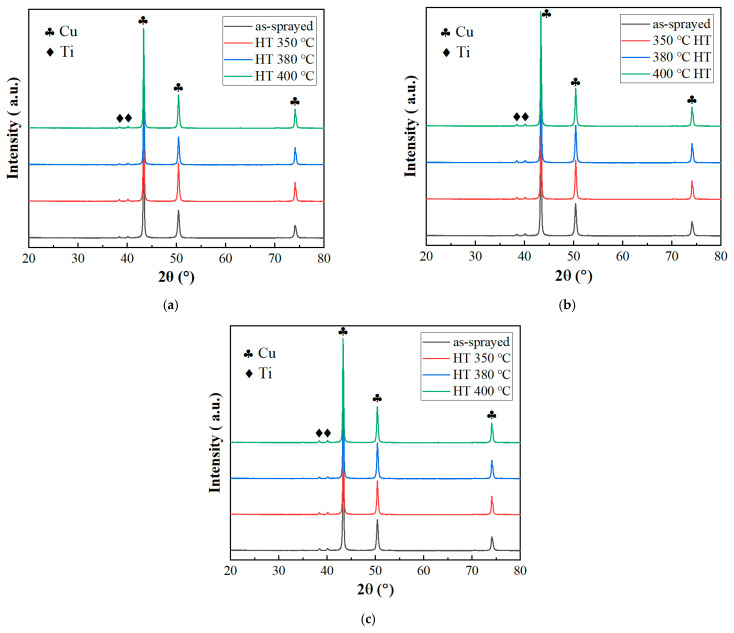
XRD patterns of cold-sprayed Cu-Ti composites before and after heat treatment under different propellant gas temperatures: (**a**) 600 °C; (**b**) 700 °C; and (**c**) 800 °C.

Figure 5 presents the XRD patterns of cold-sprayed Cu-Ti composites before and after heat treatment under propellant gas temperatures of 600 °C (a), 700 °C (b), and 800 °C (c). The as-sprayed deposits prepared at different gas temperatures exhibit highly similar diffraction peak characteristics. All samples display only Cu and Ti phases, confirming no oxidation during deposition and absence of new phase formation—indicating a purely mechanical mixture of Cu and Ti. This result aligns with findings on cold-sprayed Cu-Cr composites reported by Wu et al. [7]. After heat treatment at 350 °C, 380 °C, and 400 °C, the XRD patterns remain unchanged, retaining the exclusive presence of Cu and Ti phases. These observations demonstrate that no reaction or diffusion occurs between Cu and Ti during either cold spraying or post-heat treatment.

### 3.2. Hardness

**Figure 6:**
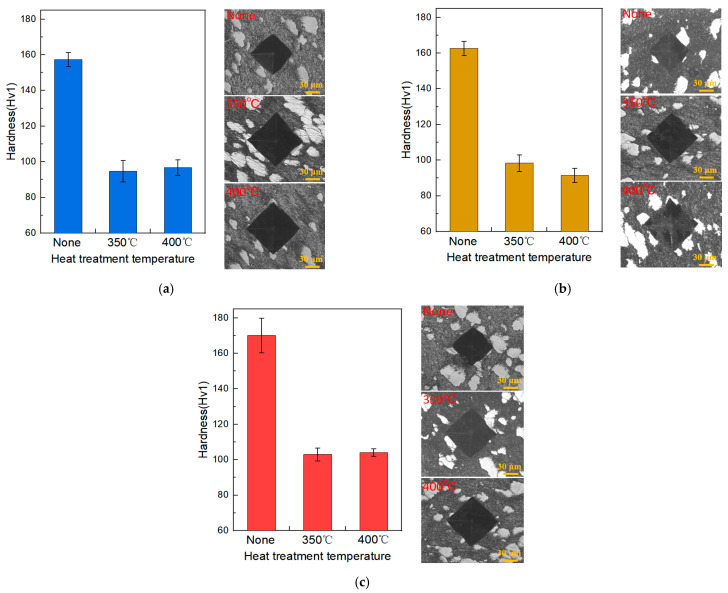
Hardness and typical indentation morphology of the Cu-Ti deposits prepared under different propellant gas temperatures before and after heat treatment: (**a**) 600 °C; (**b**) 700 °C; and (**c**) 800 °C.

The hardness results of the Cu-Ti deposits prepared with different propellant gases and those after heat treatment are presented in Figure 6. Accordingly, OM images with Vickers indentation marks are presented in Figure 6 to visually support the hardness results of the composites. The hardness of the as-prepared cold-sprayed Cu-Ti composite deposits is approximately in the range of 158 to 170 HV1. As the temperature of the propellant gas during the cold-spraying process increases, the hardness of the obtained Cu-Ti deposits increases accordingly. When the propellant gas temperature reaches 800 °C, the maximum hardness of 170.1 HV1 is achieved. This is because the increase in the propellant gas temperature leads to an increase in the gas flow velocity [23]. As a result, the Cu particles impact the substrate at a higher speed, undergoing more severe plastic deformation. This increases the internal dislocation density and forms a work-hardening effect.

By comparing the hardness of the deposits before and after heat treatment, it can be observed that the hardness of the Cu-Ti deposits prepared with different propellant gases shows a significant downward trend after heat treatment. The hardness of the deposits prepared under the propellant gas temperatures of 600 °C, 700 °C, and 800 °C decreases to 90–100 HV1 after heat treatment, with a reduction amplitude of approximately 40%. There is no significant difference in the hardness of the deposits under the two heat-treatment conditions of 350 °C and 400 °C. Li et al. [27] demonstrated that complete recrystallization occurs in cold-sprayed copper coatings under heat treatment at temperatures above 350 °C. In this study, the post-heat treatment temperature range (350–400 °C) aligns with the recrystallization temperature of cold-sprayed copper, which is known to decrease with increasing plastic deformation induced by higher propellant gas temperatures. For example, the as-sprayed deposit prepared at 800 °C, subjected to more intense particle deformation, may exhibit a slightly lower effective recrystallization temperature compared to the 600 °C counterpart. The heat treatment eliminates the work-hardening effect in the deposits, thus reducing the hardness of the deposits [28].

### 3.3. Tensile Properties

**Figure 7:**
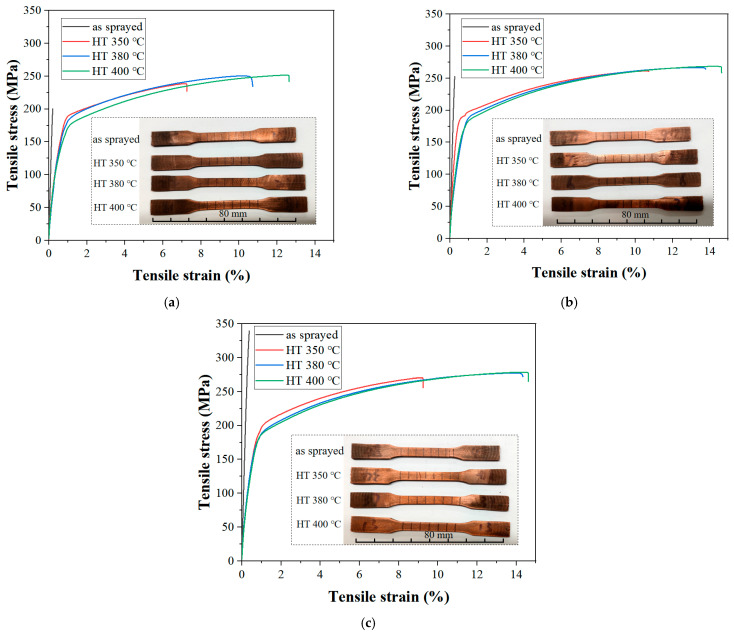
Tensile behavior of Cu-Ti deposits prepared under different propellant gas temperatures before and after heat treatment: (**a**) 600 °C propellant gas temperature: stress–strain curve (**top**) and photograph of the fractured specimen (**bottom**, aligned halves showing elongation at fracture); (**b**) 700 °C propellant gas temperature: stress–strain curve (**top**) and photograph of the fractured specimen (**bottom**, aligned halves showing elongation at fracture); (**c**) 800 °C propellant gas temperature: stress–strain curve (**top**) and photograph of the fractured specimen (**bottom**, aligned halves showing elongation at fracture).

Figure 7 shows the tensile curves of Cu-Ti composite deposits prepared under different cold-spray propellant gas temperatures (600 °C, 700 °C, 800 °C) before and after heat treatment. In the initial elastic stage of tension, each curve exhibits an approximately linear relationship between stress and strain, indicating that the material behaves elastically. The as-sprayed specimens all fracture in the elastic stage, with the tensile strain at fracture being less than 0.5%. However, the cold-spray propellant gas temperature has a significant impact on the tensile strength of the as-deposited specimens. As the propellant gas temperature increases, the strength increases.

The plasticity of the heat-treated materials is greatly improved, as clearly demonstrated by the photograph of the fractured specimen in Figure 7. The post-heat treatment specimens exhibit visibly greater elongation at fracture, with this improvement being particularly evident in the 400 °C treated samples. When the tensile strain exceeds 1%, the curves deviate from linearity and enter the plastic deformation stage. For all specimens, the tensile curves show a gently rising trend during the plastic stage. Overall, as the cold-spray propellant gas temperature increases, the strength of the Cu-Ti composite deposits increases. Post heat treatment can significantly enhance the ductility of the deposits [25]. As the post treatment temperature rises (from 350 °C to 400 °C), the strain at fracture gradually increases.

**Figure 8:**
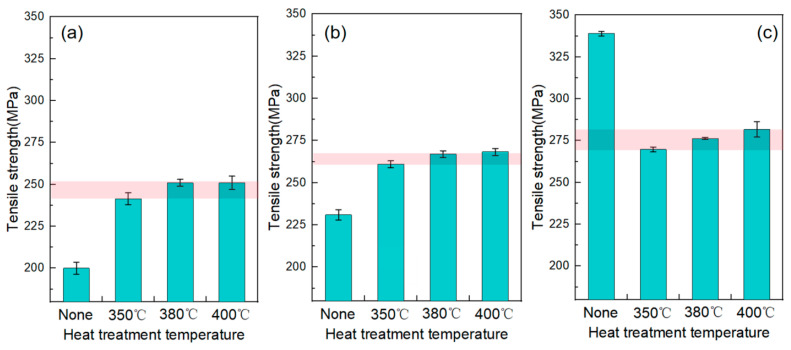
Comparison of tensile strengths of Cu-Ti deposits prepared under different propellant gas temperatures before and after heat treatment: (**a**) 600 °C; (**b**) 700 °C; and (**c**) 800 °C.

Figure 8 presents a comparison of the specific tensile strengths of Cu-Ti composite deposits under different heat treatment temperatures, with cold-spray propellant gas temperatures of 600 °C, 700 °C, and 800 °C. For the deposits obtained at a propellant gas temperature of 600 °C, the tensile strength of the as-deposited material is approximately 200 MPa. However, after heat treatment at 350 °C, it significantly increases to around 240 MPa. When the heat-treatment temperature is further increased to 380 °C and 400 °C, the tensile strength of the material reaches about 250 MPa.

For the Cu-Ti deposits obtained at a propellant gas temperature of 700 °C, the tensile strength of the as-sprayed deposit is approximately 230 MPa. After heat treatment, the tensile strength increases to 260–270 MPa. The increase in the tensile strength of the deposits after heat treatment is mainly due to the enhanced inter-particle bonding within the deposits under the action of high temperature [25].

For the Cu-Ti deposits obtained at a propellant gas temperature of 800 °C, the as-sprayed deposit has a relatively high tensile strength, reaching 338 MPa. After heat treatment, the tensile strength decreases compared to the as-sprayed state, being approximately 270–280 MPa, slightly higher than that of the deposits obtained at a propellant gas temperature of 700 °C after heat treatment.

It can be observed that as the cold-spray propellant gas temperature increases, the tensile strength of the as-prepared deposits shows a significant upward trend. A Cu-Ti composite material with a strength of 338 MPa is obtained at a propellant gas temperature of 800 °C. This is because, with the propellant gas at a higher temperature, copper particles are accelerated more significantly, leading to more intense deformation upon impact during the formation of the deposited coating. The bonding between particles is enhanced, and the strength is improved due to the work-hardening effect [16].

While the propellant gas temperature in cold spraying significantly affects the strength of as-deposited Cu-Ti composite coatings, this parametric dependency is notably reduced after post-deposition heat treatment. Comparative analysis shows that coatings prepared at 800 °C propellant gas temperature exhibit only a moderate 30 MPa (12%) strength increase compared to those prepared at 600 °C after the same thermal treatment. Additionally, a systematic assessment of heat-treatment parameters reveals little effect on the ultimate tensile strength within the investigated temperature range. Although increasing the annealing temperature from 350 °C to 400 °C produces a measurable strength improvement, the observed marginal 4% increase indicates limited process sensitivity in this range.

Compared with cold-sprayed pure copper, the Cu-Ti composite prepared in this study exhibits a significant enhancement in tensile strength. According to the research by Boyle et al. [29], the as-sprayed tensile strength of cold-sprayed pure copper at 600 °C is 170 MPa, which increases to 190 MPa after heat treatment at 350 °C. In contrast, the Cu-Ti composite processed by 800 °C spraying followed by 400 °C heat treatment achieves a tensile strength of 270 MPa, representing a 42% increase compared to the heat-treated pure copper. Additionally, the tensile strength of traditionally wrought pure copper is only 194 MPa [30], further validating the effectiveness of combining cold spray processing with Ti reinforcement for strength optimization.

**Figure 9:**
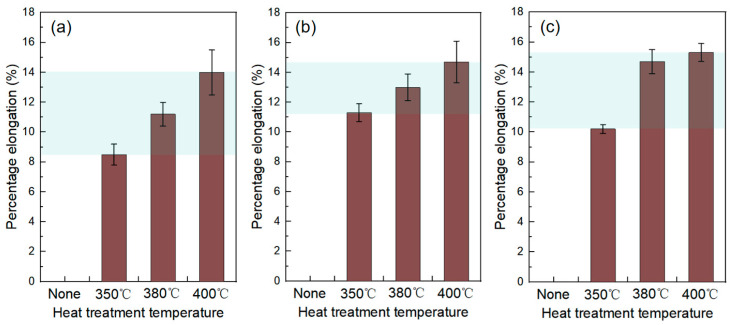
Comparison of percentage elongation after fracture of Cu-Ti deposits prepared under different propellant gas temperatures before and after heat treatment: (**a**) 600 °C; (**b**) 700 °C; and (**c**) 800 °C.

Figure 9 shows a comparison of the percentage elongation after fracture of Cu-Ti deposits prepared under different propellant gas temperatures (600 °C, 700 °C, 800 °C) before and after heat treatment. All as-sprayed deposits fractured without effective deformation, exhibiting typical brittle fracture characteristics. However, after heat treatment, significant plastic deformation occurred during the tensile process of the deposits, with the percentage elongation ranging from 8.5% to 15%. This is mainly because recrystallization and grain growth occur at higher temperatures, and heat treatment eliminates work-hardening, thereby improving the ductility of the material [25].

Both the cold-spray propellant gas temperature and the post heat treatment temperature have a significant impact on the percentage elongation of Cu-Ti deposits. For the deposits prepared by spraying under a propellant gas temperature of 600 °C, the percentage elongation is 8.5% after heat treatment at 350 °C. As the temperature increases, the percentage elongation gradually rises, reaching 14% after heat treatment at 400 °C. Deposits prepared by spraying under propellant gas temperatures of 700 °C and 800 °C also exhibit similar patterns. Moreover, as the propellant gas temperature increases, the percentage elongation of the deposits after the same heat treatment is higher than that of deposits prepared at lower propellant gas temperatures.

The results of the tensile tests indicate that the tensile properties of Cu-Ti composites prepared by cold-spray additive manufacturing can reach a tensile strength of 270 MPa and a percentage elongation of 15% after heat treatment.

### 3.4. Analysis of Tensile Fracture Behavior

**Figure 10:**
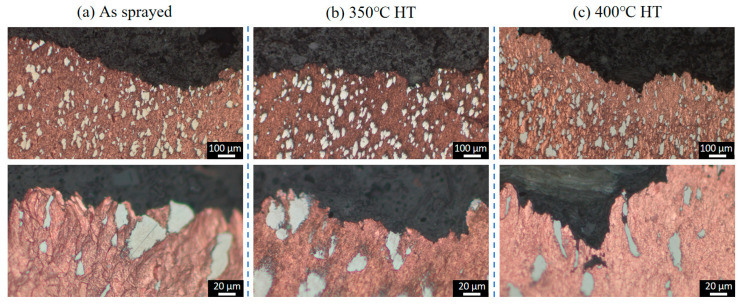
Metallographic of the fracture cross-sections of the Cu-Ti deposits prepared by cold spraying under the propellant gas temperature condition of 600 °C before and after heat treatment: (**a**) as-sprayed state; (**b**) 350 °C heat treatment; and (**c**) 400 °C heat treatment.

**Figure 11:**
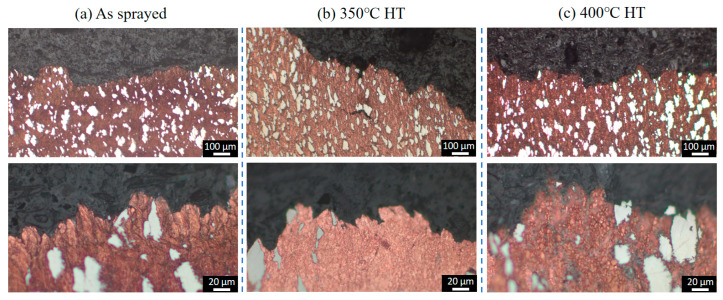
Metallographic of the fracture cross-sections of the Cu-Ti deposits prepared by cold spraying under the propellant gas temperature condition of 700 °C before and after heat treatment: (**a**) as-sprayed state; (**b**) 350 °C heat treatment; and (**c**) 400 °C heat treatment.

**Figure 12:**
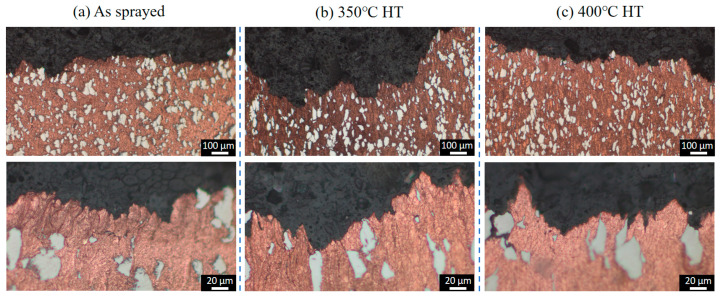
Metallographic of the fracture cross-sections of the Cu-Ti deposits prepared by cold spraying under the propellant gas temperature condition of 800 °C before and after heat treatment: (**a**) as-sprayed state; (**b**) 350 °C heat treatment; and (**c**) 400 °C heat treatment.

Figure 10, Figure 11 and Figure 12 present the cross-sectional microstructures of tensile fracture surfaces for Cu-Ti deposits prepared at propellant gas temperatures of 600 °C, 700 °C, and 800 °C, comparing as-sprayed and heat-treated conditions. All deposits exhibited similar fracture characteristics: fractures predominantly occurred within the copper particle matrix, with no observed fracture of titanium particles. Magnified views near fracture surfaces revealed significant microstructural differences in copper between as-sprayed and heat-treated states. The as-sprayed specimens displayed typical cold-sprayed lamellar particle stacking, while post-heat treatment specimens showed refined grain structures due to copper recrystallization. Notably, subsurface crack propagation was observed beneath fracture surfaces in 600 °C and 700 °C specimens, but absent in 800 °C specimens. This demonstrates that deposits fabricated with higher propellant gas temperatures possess enhanced cohesive bonding strength, consequently achieving superior tensile properties. These findings align with the trends observed in cold-sprayed pure copper systems under analogous processing conditions [31].

**Figure 13:**
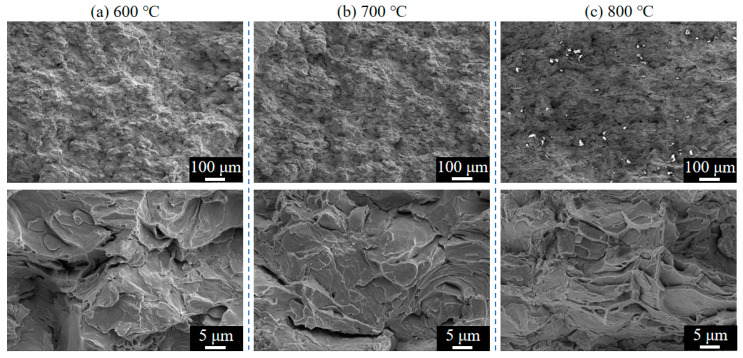
Fracture surface morphologies of as-sprayed Cu-Ti composite deposits prepared at different propellant gas temperatures: (**a**) 600 °C; (**b**) 700 °C; and (**c**) 800 °C.

Fractographic analysis provides direct visualization of material fracture behavior. Figure 13 illustrates the tensile fracture morphologies of as-sprayed Cu-Ti deposits fabricated at propellant gas temperatures of 600 °C, 700 °C, and 800 °C. All specimens exhibited cleavage brittle fracture patterns while retaining the typical cold-sprayed particle stacking features. Microscopic observations revealed a progressive thinning of deformed flattened particles with increasing gas temperature: discrete particle morphology predominated at 600 °C, whereas distinct lamellar sheet structures became prominent at 800 °C. This morphological evolution demonstrates the critical role of propellant gas temperature in governing particle deformation degree. Under elevated temperature conditions (800 °C), particles underwent substantial plastic deformation, which not only induced notable work-hardening effects but also enhanced interparticle bonding through increased contact area and metallurgical bonding strength, thereby significantly improving the overall tensile strength of the deposits.

**Figure 14:**
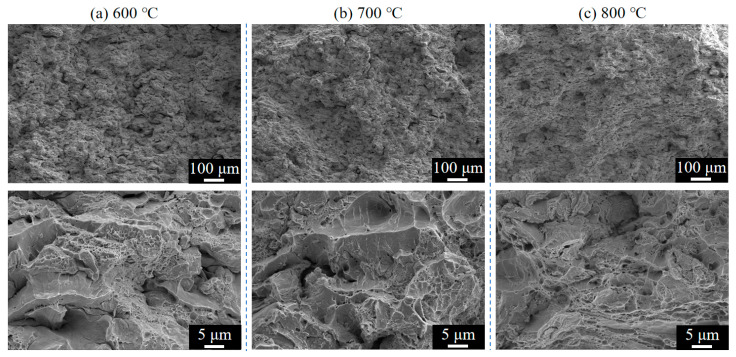
Fracture surface morphologies of Cu-Ti composite deposits prepared at different propellant gas temperatures after 350 °C heat treatment: (**a**) 600 °C; (**b**) 700 °C; and (**c**) 800 °C.

**Figure 15:**
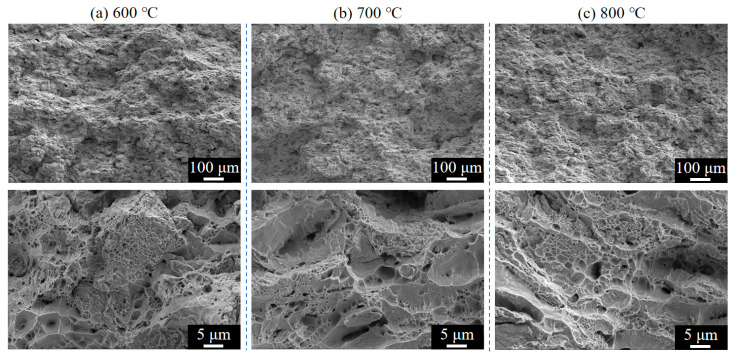
Fracture surface morphologies of Cu-Ti composite deposits prepared at different propellant gas temperatures after 400 °C heat treatment: (**a**) 600 °C; (**b**) 700 °C; and (**c**) 800 °C.

Figure 14 and Figure 15 present the fracture surface morphologies of Cu-Ti composite deposits prepared at varying propellant gas temperatures and subsequently heat-treated at 350 °C (Figure 14) and 400 °C (Figure 15). All heat-treated specimens exhibit pronounced dimple aggregation on tensile fracture surfaces, indicative of ductile fracture behavior, which aligns with the plastic deformation features observed in stress–strain curves. Comparative analysis reveals a temperature-dependent trend in dimple density: specimens treated at 350 °C display mixed ductile–brittle characteristics, whereas those treated at 400 °C exhibit dominant microvoid coalescence. Notably, the specimen processed at 800 °C propellant gas and under 400 °C heat treatment demonstrates optimal ductility, characterized by high-density, uniformly distributed, fine equiaxed dimples. This confirms that 400 °C heat treatment effectively enhances ductility while preserving tensile strength.

## 4. Conclusions

This study fabricated Cu-Ti composite deposits via cold spray technology under varying propellant gas temperatures, systematically investigating the effects of gas temperature and post-heat treatment on their microstructures and mechanical properties. The key findings are as follows:

(1) The cold-spray propellant gas temperature significantly regulates the properties of Cu-Ti deposits. As the temperature increases from 600 °C to 800 °C, the tensile strength of the deposits improves, reaching 338 MPa at 800 °C, which is attributed to the plastic deformation and work-hardening effect caused by high-speed particle impact. Post-heat treatment promotes copper matrix recrystallization, eliminates work hardening, and increases the elongation to 15% after 400 °C heat treatment.
(2) Fracture microanalysis reveals that a high propellant gas temperature enhances the deposits’ cohesive strength. Subsurface cracking was observed beneath fracture surfaces in specimens prepared at 600 °C and 700 °C, while absent in 800 °C specimens, highlighting the advantage of higher propellant gas temperatures in improving cohesive bonding strength.
(3) The optimized process combination (800 °C propellant gas + 400 °C heat treatment) achieves synergistic property optimization. The deposit exhibits a tensile strength of 270 MPa and an elongation of 15%, providing key process references for the application of cold-sprayed Cu-Ti composites in the engineering field.

## Data Availability

The original contributions presented in this study are included in the article. Further inquiries can be directed to the corresponding authors.
